# Occupational stress, coping and mental health in Jamaican police officers

**DOI:** 10.1093/occmed/kqw055

**Published:** 2016-04-30

**Authors:** K. V. Nelson, A. P. Smith

**Affiliations:** Centre for Occupational and Health Psychology, School of Psychology, Cardiff University, Cardiff CF10 3AS, UK.

**Keywords:** Coping, mental health, occupational stress, police officers.

## Abstract

**Background:**

Police are exposed to a wide range of stressors and this is especially true in developing countries such as Jamaica. Exposure to psychosocial stressors and use of maladaptive coping styles can result in mental ill-health.

**Aims:**

To examine the relationship between work characteristics, coping and mental health in Jamaican police officers and to test whether work characteristics are indirectly associated with mental health outcomes through perceived job stress and job satisfaction.

**Methods:**

Police officers from the Jamaican police force completed a questionnaire using a cross-sectional design. We analysed the data using hierarchical regression.

**Results:**

The study group consisted of 134 police officers; the response rate was 94%. Negative work characteristics, lower levels of positive work factors and work support and emotion-focused coping styles were associated with increased levels of depression (*F*(8, 125) = 7.465, *P* < 0.001). Subjective feelings of anxiety were positively associated with negative work characteristics and emotion-focused coping (*F*(8, 125) = 7.586, *P* < 0.001). The relationship between work characteristics and mental health outcomes was mediated by perceived stress. Job satisfaction mediated the relationship between positive work characteristics and depression.

**Conclusions:**

Stress management and intervention programmes should address modifiable work conditions, monitor stress levels and reduce maladaptive coping.

## Introduction

Police officers are a vulnerable group for mental ill-health [1,2]. However, police studies have largely adopted a stressor-strain approach. Researchers argue that this is an overly simplistic approach as it fails to include import ant individual differences such as coping styles and subjective appraisals [3]. Empirical evidence on the direct effect of coping strategies on well-being is unclear. Some police studies show that maladaptive coping leads to adverse health outcomes, whereas others found the influence of coping on mental health outcomes to be negligible [2,4]. Subjective appraisals such as perceived job stress can mediate the relationship between work characteristics and health outcomes [5]. However, few police stress studies incorporate mechanisms by which subject ive appraisals can impact the relationship between work conditions and outcomes [6]. Existing research may not be generalizable to Jamaica, where officers work in harsh policing environments characterized by the high volume and violent nature of crime [7]. This study sought to investigate the relationships between work characteristics, coping styles and mental health in Jamaican police and to determine whether work characteristics indirectly influence mental health through job stress and job satisfaction.

## Methods

Ethical approval for this study was obtained from the School of Psychology at Cardiff University and consent for conducting the study was obtained from the Commissioner of the Jamaican Police Service. We approached three groups of police officers who were participating in developmental training courses. These groups were selected based on ease of access around the time of data collection. Training coordinators allowed for the distribution and collection of questionnaires prior to the start of classes.

The questionnaire included measures of demographic details, work characteristics, coping, perceived stress, job satisfaction and mental health outcomes. We used single items from the Well-being Process Questionnaire (WPQ), which have been validated against multi-items measures, and shown to perform just as well as full-length measures with which they are compared [8]. The WPQ has been shown to perform well in measuring well-being in other occupational groups [8,9].

We used hierarchical regression to test the relative effect of groups of predictors on outcome. We conducted simple mediation analyses using the Hayes PROCESS tool for SPSS. Mediation is said to occur when the confidence interval for indirect effect does not contain zero [10].

## Results

We distributed questionnaires to 142 police officers from the Jamaican Police Service and 134 participated in the study (response rate 94%). The mean age of the sample was 32 years (SD = 6.53) and the mean years of service 9 years (SD =5.95). Most participants were men (63%); 48% were constables, 20% corporals, 30% sergeants and 2% inspectors.

Principal components analyses revealed three factors for work characteristics: negative work characteristics (i.e. demand, effort, consultation on change, over-commitment and role understanding); positive work characteristics (i.e. reward and control) and work support [i.e. colleague support, supervisor support, supervisor relationship and bullying (recoded)]. Two coping factors emerged: emotion-focused coping (self-blame, wishful thinking and avoidance) and action-oriented coping (problem-focused and seek social support). We used these factors in subsequent analyses.

Each block of predictors, except for demographic vari ables, made a significant contribution to the outcomes (Table 1). Work factors entered in the second block made a significant overall and individual contribution to depression. Although as a whole work factors contributed significantly to anxiety, this was largely accounted for by the significant influence of negative work characteristics. Coping styles on the whole accounted for a significant increase in variance for both outcomes but only emotion-focused coping had a significant individual effect. The variables explained 32 and 33% of variance in depression and anxiety, respectively.

**Table 1.** Standardized regression coefficients for demographic variables, work characteristics and coping as predictors of depression and anxiety

	Depression			Anxiety		
	Step 1 β	Step 2 β	Step 3 β	Step 1β	Step 2 β	Step 3 β
Control variables						
Gender	0.13	0.12	0.06	0.09	0.09	−0.02
Years of service	0.00	−0.00	0.03	0.02	0.00	0.05
Rank	−0.04	0.05	−0.01	−0.06	−0.00	−0.08
Work characteristics						
Negative work characteristics		0.32***	0.24**		0.36***	0.23**
Positive work characteristics		−0.25**	−0.21*		−0.13	−0.05
Work support		−0.29***	−0.26***		−0.11	−0.03
Coping						
Emotion-focused coping			0.26**			0.43***
Action-oriented coping			−0.04			−0.14
*F*	0.894	7.678***	7.465***	0.476	4.186***	7.586***
*R* ^2^	0.020	0.266	0.323	0.011	0.165	0.327
*R* ^2^Δ		0.246***	0.057**		0.154***	0.162***

Gender: male = 0, female = 1. Rank: constable = 0, above constable = 1.

*P < 0.05.

**P < 0.01.

***P < 0.001.

All three work factors indirectly influenced depression and anxiety through perceived stress. Positive work characteristics indirectly influenced depression through job satisfaction (Table 2).

**Table 2.** Mediating effects of perceived job stress and job satisfaction on the relationship between work characteristics and mental health

	Total effects	Direct effects	Indirect effects
Depression			
Mediator: perceived stress			
Negative work characteristics	*b* = 0.723, CI 0.344 to 1.102 (*P* < 0.001)	*b* = 0.581, CI 0.184 to 0.979 (*P* < 0.01)	*b* = 0.142, CI 0.028 to 0.339^a^
Positive work characteristics	*b* =−0.631, CI −1.015 to −0.246 (*P* < 0.001)	*b* = −0.486, CI −0.884 to −0.088 (*P* < 0.05)	*b* = −0.145, CI −0.334 to −0.029^a^
Work support	*b* = −0.689, CI −1.078 to −0.299 (*P* < 0.001)	*b* = −0.558, CI −0.956 to −0.160 (*P* < 0.01)	*b* = −0.131, CI −0.320 to −0.030^a^
Mediator: job satisfaction			
Negative characteristics	*b* = 0.725, CI 0.344 to 1.105 (*P* < 0.001)	*b* = 0.737, CI 0.376 to 1.098 (*P* < 0.001)	*b* = −0.606, CI −0.270 to 0.008
Positive work characteristics	*b* = −0.631, CI −1.017 to −0.244 (*P* < 0.01)	*b* = −0.432, CI −0.838 to −0.027 (*P* < 0.05)	*b* = −0.198, CI −0.416 to −0.066^a^
Work support	*b* = −0.697, CI −1.092 to −0.302 (*P* < 0.01)	*b* = −0.606, CI −0.991 to −0.221 (*P* < 0.01)	*b* = −0.091, CI −0.270 to 0.008
Anxiety			
Mediator: perceived stress			
Negative work characteristics	*b* = 0.840, CI 0.460 to 1.219 (*P* < 0 .001)	*b* = 0.639, CI 0.250 to 1.028 (*P* < 0.001)	*b* = 0.201, CI 0.073 to 0.398^a^
Positive work characteristics	*b* = −0.357, CI −0.757 to 0.043 (NS)	*b* = −0.117, CI −0.517 to 0.283 (NS)	*b* = −0.240, CI −0.477 to −0.106^a^
Work support	*b* = −0.229, CI −0.639 to 0.182 (NS)	*b* = 0.003, CI −0.400 to 0.407 (NS)	*b* = −0.232, CI −0.458 to −0.094^a^
Mediator: job satisfaction			
Negative work characteristics	*b* = 0.839, CI 0.458 to 1.220 (*P* < 0.001)	*b* = 0.837, CI 0.454 to 1.219 (*P* < 0.001)	*b* = 0.002, CI −0.028 to 0.072
Positive work characteristics	*b* = −0.361, CI −0.764 to 0.041 (NS)	*b* = −0.454, CI −0.885 to −0.024 (*P* < 0.05)	*b* = 0.093, CI −0.093 to 0.295
Work support	*b* = −0.240, CI −0.656 to 0.176 (NS)	*b* = −0.260, CI −0.681 to 0.162 (NS)	*b* = 0.020, CI −0.038 to 0.139

CI, confidence interval; NS, non-significant.

^a^Significant indirect effects.

## Discussion

Our findings indicate that negative work characteristics and emotion-focused coping were associated with high levels of depression and anxiety. Positive work characteristics and work support were inversely related to depression but not associated with anxiety. All three work factors indirectly influenced mental health through perceived stress. Job satisfaction partially mediated the relationship between positive work characteristics and depression.

To our knowledge, this is the first study to examine perceived stress and job satisfaction in mediating the relationship between work conditions and mental health in police. However, the study has some limitations, which should be considered when interpreting findings. These include the cross-sectional design, small sample and single-item self-report measures. These may introduce bias and limit our ability to infer causal relationships and generalize to the general police population and other police organizations.

Our findings are consistent with previous research that found adverse working conditions, low levels of positive work factors and poor relationships with peers and supervisors to be associated with depressive symptoms. Weaker associations were found with anxiety and work characteristics, which have also been demonstrated in one study measuring similar variables [5]. Our findings support the importance of emotion-focused coping in predicting depression and anxiety, though problem-focused coping did not show a significant influence. This is consistent at least in part with previous research [3]. Studies have demonstrated the importance of not only considering the work stressor itself but also how subjective appraisals of work conditions can influence health outcomes [6]. Our findings support this research framework in the context of police stress research.

Despite the limitations, our study serves as a starting point for future studies in a population with no published research. The Jamaican police service may consider these findings useful for targeted interventions. This may involve periodically monitoring and auditing stress levels and job satisfaction while improving organizational practices such as support to reduce emotional coping. Future research should include larger samples and longitudinal methods to test the robustness of these findings. Additionally, a multi dimensional approach including additional variables such as personality and work–life interface should be included with an assessment of mediation and moderation effects.

**Key points**

- Negative work characteristics and emotion-focused coping were positively associated with depression and anxiety in Jamaican police officers.
- Positive work characteristics and work support were inversely associated with depression but were not significantly associated with anxiety.
- Work characteristics indirectly influenced mental health through perceived stress.

## Funding

Commonwealth Scholarship Commission (to K.V.N.).

## Conflicts of interest

None declared.
